# Wheat Bran Extract Regulates Mast Cell-Mediated Allergic Responses In Vitro and In Vivo

**DOI:** 10.3390/molecules25173997

**Published:** 2020-09-02

**Authors:** Jae Yeon Lee, Eun-Kyung Ahn, Ju-Hyoung Park, Joa Sub Oh

**Affiliations:** 1 Bio-Center, Gyeonggido Business and Science Accelerator, Gwanggyo-ro 147, Yeoungtong, Suwon, Gyeonggi 16229, Korea; jyeon@gbsa.or.kr (J.Y.L.); aek@gbsa.or.kr (E.-K.A.); 2 College of Pharmacy, Dankook University, Dandae-ro 119, Dongnam, Cheonan, Chungnam 31116, Korea; yourselves@naver.com

**Keywords:** wheat bran, mitogen-activated protein kinase, allergy, anaphylaxis

## Abstract

In the present study the effects and molecular mechanisms of wheat bran (WB), the hard outer layer of the wheat kernel used in food ingredients, on mast cell-mediated allergic responses in vitro and in vivo were investigated. The water extract of WB inhibited degranulation and expression of allergic and inflammatory mediators such as tumor necrosis factor-α, cyclooxygenase-2 and inducible nitric oxide synthase in antigen-stimulated RBL-2H3 cells. These anti-allergic activities of WB were mediated by the inactivation of extracellular signal-regulated kinase and p38 mitogen-activated protein kinase, which play important roles in degranulation and expression of various allergic and inflammatory molecules. In agreement with its in vitro effects, WB inhibited immunoglobulin E (IgE)/antigen-induced and compound 48/80-induced anaphylactic reactions in vivo. Taken together, these findings suggest the pharmacological potential of WB in the regulation of allergic diseases, including allergic rhinitis, atopic dermatitis, asthma and anaphylaxis.

## 1. Introduction

Allergic diseases such as atopic dermatitis, allergic rhinitis and asthma are induced by hypersensitive immune reactions to normally harmless substances (allergens) in the environment, foods or drugs [1,2,3]. These over-reactive, inappropriate immune responses are associated with genetic and/or environmental factors [3,4]. Mast cells play important roles in immunoglobulin E (IgE)-mediated allergy and anaphylaxis in response to allergen exposure. Allergen-mediated aggregation of IgE high affinity receptors (FcεRΙ) on mast cells triggers the degranulation and secretion of various allergic mediators such as histamine, inflammatory cytokines and enzymes, suggesting the rational strategy and pharmacological efficacy of FcεRΙ signaling pathway-targeted therapeutics for the treatment of acute and chronic allergic reactions [5,6]. Various clinical approaches for patients with allergic diseases include the treatment with anti-histamines, nasal decongestants, corticosteroids, IgE blockers, or cytokine-based immunotherapy as well as avoidance of allergens; however, there has been only modest progress in improving clinical outcomes and lowering side effects [7,8,9]. Thus, complementary and alternative medicines are appreciated as one of the potent and promising strategies for the development of anti-allergic therapeutics.

Wheat bran (WB), the hard outer layer of the wheat kernel, is used as a common ingredient in foods and contains various nutrients, minerals and phytochemicals as well as insoluble dietary fibers [10,11]. These components of WB have been known to exert beneficial biological activities including anti-oxidative, nutrition and prebiotic activities [11,12,13]. Many studies indicate that WB may help improve the health and prevent the occurrence and progression of chronic pathologic conditions such as gastrointestinal inflammation, cancer, cardiovascular and metabolic diseases [14,15,16,17]. However, the effects and molecular mechanisms of WB on allergic responses are poorly understood. Therefore, the present study aims to determine the effects and underlying mechanisms of the water extract and bioactive components from WB on mast cell-mediated allergic responses in vitro and in vivo.

## 2. Results

### 2.1. Effect of WB on Antigen-Induced Degranulation in RBL-2H3 Cells

**Figure 1:**
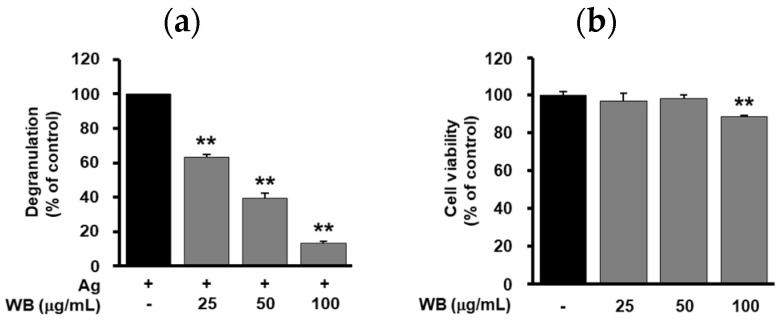
Effects of WB on degranulation and viability in RBL-2H3 cells. (**a**) Cells were pretreated with WB (25–100 μg/mL) for 20 min, followed by antigen stimulation for 20 min. Degranulation was determined by measuring the activity of β-hexosaminidase as described in Materials and Methods. (**b**) Cells were treated with WB (25–100 μg/mL) for 24 h. Cell viability was determined using MTT assay. The results from triplicate determinations are presented as the percentage of maximal antigen-induced degranulation or that of viable cells. Statistical significance is indicated (** *p* < 0.01, compared with (**a**) antigen-treated cells or (**b**) untreated controls).

Mast cell-derived secretory mediators such as histamine, cytokines and various enzymes play important roles in allergic responses [18]. Thus, the ability of WB to regulate the release of β-hexosaminidase, a degranulation marker of mast cell activation, was first examined. As shown in Figure 1a, WB treatment dose-dependently inhibited antigen-induced degranulation in RBL-2H3 cells (IC_50_ 41.35 μg/mL). Following treatment with WB for 24 h, cell viability was determined by MTT assay. Since marginal or weak cytotoxicity was observed at the highest concentration (100 μg/mL), in the next study the effects of WB on allergic responses in vitro were examined at concentrations below 100 μg/mL (Figure 1b).

### 2.2. Effect of WB on Antigen-Induced Expression of Allergic and Inflammatory Mediators in RBL-2H3 Cells

**Figure 2:**
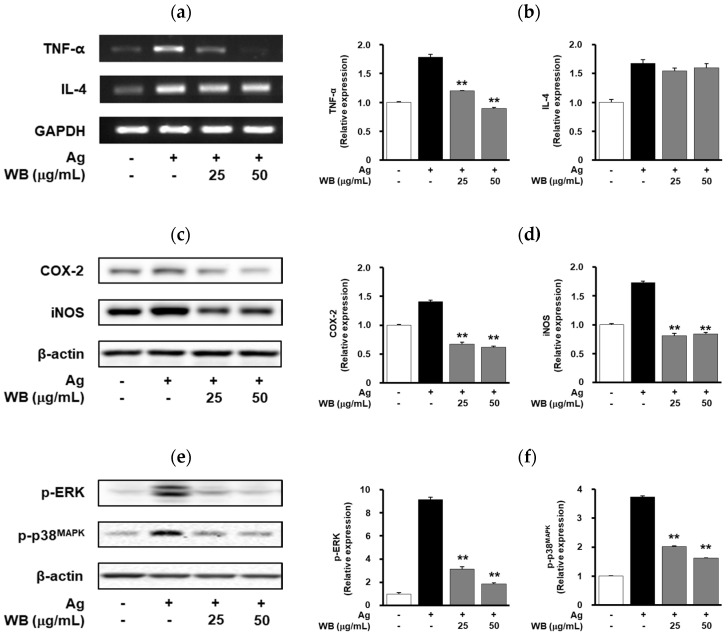
Effects of WB on allergic mediators and MAPK signaling pathway. RBL-2H3 cells were pretreated with WB (25, 50 μg/mL) for 20 min, followed by antigen stimulation for 20 min. The levels of TNF-α, IL-4, COX-2, iNOS, p-ERK and p-p38^MAPK^ were determined by (**a**) RT-PCR and (**c**,**e**) Western blot analyses. Results shown are representative of at least three independent experiments. (**b**,**d**,**f**) Integrated density values were normalized to untreated controls. Statistical significance is indicated (** *p* < 0.01, compared with antigen-treated cells).

Some inflammatory cytokines including tumor necrosis factor-α (TNF-α) and interleukin-4 (IL-4) are mainly involved in the pathogenesis of allergic and inflammatory diseases [19]. WB treatment markedly inhibited antigen-induced expression of TNF-α to levels observed in untreated controls (Figure 2a,b). In contrast, the levels of IL-4 were not altered by WB treatment. In addition, WB treatment inhibited antigen-induced expression of cyclooxygenase-2 (COX-2) and inducible nitric oxide synthase (iNOS) in a dose-dependent manner (Figure 2c,d). To investigate the molecular mechanisms and targets of WB in regulating mast cell activation, we analyzed the activation of mitogen-activated protein kinases (MAPKs) such as extracellular signal-regulated kinase (ERK) and p38 mitogen-activated protein kinase (p38^MAPK^), which are closely associated with degranulation and secretion of various inflammatory mediators [20]. WB treatment markedly inhibited the phosphorylation of ERK and p38^MAPK^ in antigen-stimulated cells (Figure 2e,f). Collectively, these findings indicate that WB negatively regulates the antigen-induced production of inflammatory mediators through the inhibition of FcεRΙ-MAPK signaling pathways.

### 2.3. Effect of WB on IgE/Antigen-Induced Passive Cutaneous Anaphylaxis (PCA) in Rats

**Figure 3:**
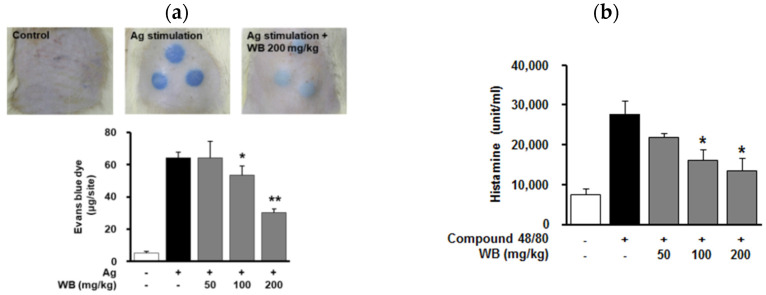
Effect of WB on IgE-induced PCA and compound 48/80-induced degranulation. (**a**) DNP-specific IgE was intradermally injected into rat dorsal skin. After 48 h, WB (50–200 mg/kg) was orally administered 1 h prior to intravenous injection with the antigen DNP-BSA containing Evans blue. Amount of dye extravasated by antigen stimulation was determined as described Materials and Methods. Results shown are representative of at least three independent experiments (*n* = 5 for each group). Statistical significance is indicated (* *p* < 0.05, ** *p* < 0.01, compared with antigen treatment group). (**b**) WB (50–200 mg/kg) was orally administered 1 h prior to intraperitoneal injection with compound 48/80 into mice. The levels of histamine were determined using a fluorometric method as described Materials and Methods. Statistical significance is indicated (* *p* < 0.05, compared with compound 48/80 treatment group).

Based on previous findings that WB inhibits mast cell degranulation and expression of allergic mediators, the effects of WB on the IgE-dependent, mast cell-driven allergic skin responses in vivo were next evaluated. As anticipated, the sequential injection of IgE and antigen DNP-BSA in rats markedly induced IgE/antigen-mediated PCA reaction. In contrast, WB treatment inhibited antigen-induced PCA reaction in a dose-dependent manner (Figure 3a).

### 2.4. Effect of WB on Compound 48/80-Induced Systemic Anaphylaxis in Mice

In vitro and in vivo anti-allergic activities of WB led us to investigate the effect of WB on compound 48/80-induced lethal systemic immune reactions. Following intraperitoneal injection of compound 48/80, we monitored the mortality rate for 1 h. As shown in Table 1, all mice injected with compound 48/80 died within 1 h, whereas WB treatment at the highest concentration (200 mg/kg) completely inhibited compound 48/80-induced mortality. Furthermore, WB treatment dose-dependently inhibited compound 48/80-induced release of histamine, one of the key allergic mediators associated with immediate phase of lethal anaphylaxis (Figure 3b). These findings suggest that WB exerts anti-allergic activity against compound 48/80-induced systemic anaphylaxis through the inhibition of histamine release.

### 2.5. Effect of WB-Derived Compounds on the Release of β-Hexosaminidase and Expression of TNF-α in Antigen-Stimulated RBL-2H3 Cells

**Figure 4:**
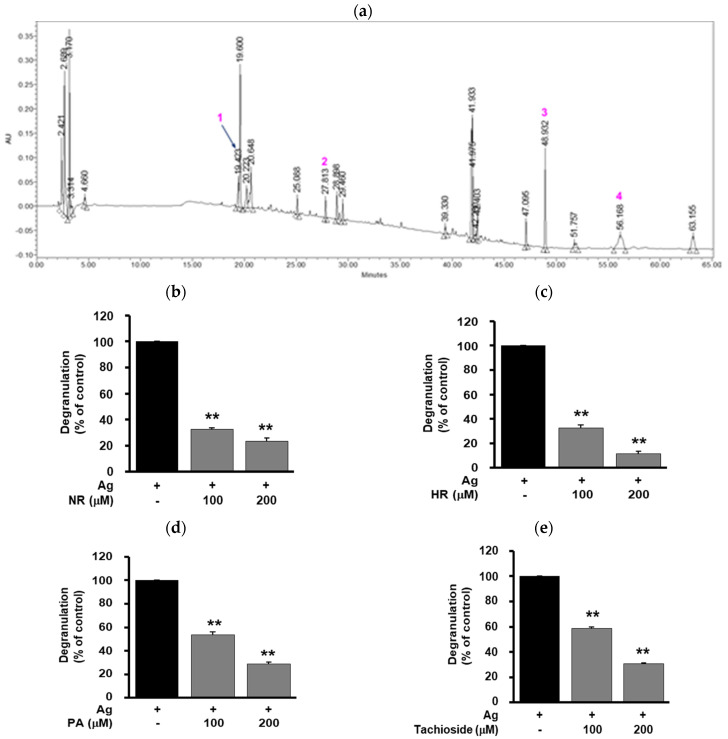
Effect of WB-derived compounds on degranulation. (**a**) HPLC chromatogram of the water extract of WB. Compounds were identified as tachioside (1), pinellic acid (2, PA), 5-*n*-nonadecylresorcinol (3, NR) and 5-*n*-heneicosylresorcinol (4, HR). (**b**–**e**) Cells were pretreated with (**b**) NR, (**c**) HR, (**d**) PA or (**e**) tachioside (100, 200 μM) for 20 min, followed by antigen stimulation for 20 min. The results from triplicate determinations are presented as the percentage of maximal antigen-induced degranulation. Statistical significance is indicated (** *p* < 0.01, compared with antigen-treated cells).

It has been reported that mast cell degranulation-inhibitory compounds from the ethanol extract of WB were isolated by bioassay-guided separation techniques [21]. In order to identify the compounds of the water extract of WB that contribute to anti-allergic activity, the chemical profile of the water extract was analyzed using HPLC. Several distinctive peaks in HPLC chromatogram of the water extract of WB were identified by comparing of ^1^H-, ^13^C-NMR and MS data with previous reports: 1 (t_R_ = 19.4 min), tachioside; 2 (t_R_ = 27.8 min), pinellic acid; 3 (t_R_ = 48.9 min), 5-*n*-nonadecylresorcinol; 4 (t_R_ = 56.2 min), 5-n-heneicosylresorcinol (Figure 4a) [21,22,23,24].

**Figure 5:**
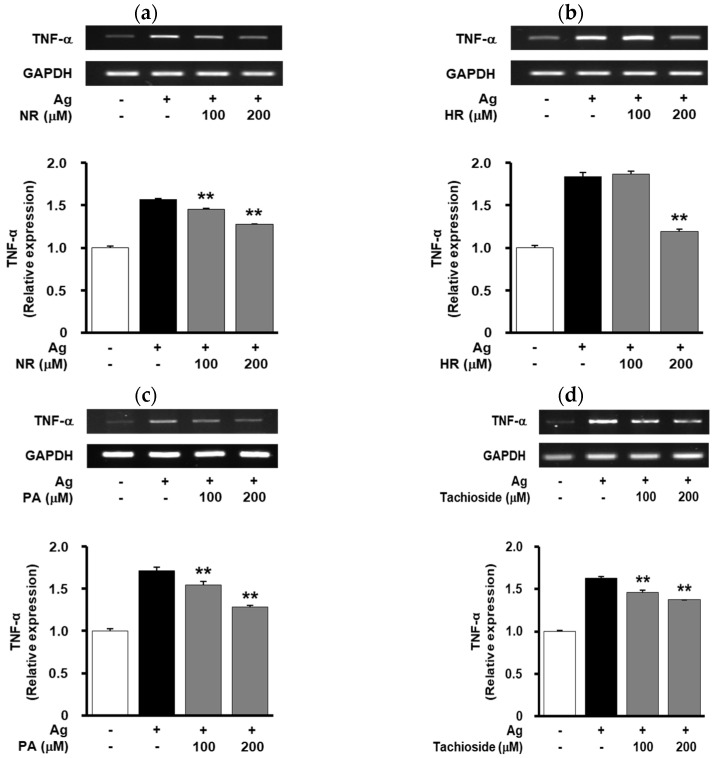
Effect of WB-derived compounds on TNF-α expression. Cells were pretreated with (**a**) NR, (**b**) HR, (**c**) PA or (**d**) tachioside (100, 200 μM) for 20 min, followed by antigen stimulation for 20 min. The levels of TNF-α were determined by RT-PCR. Results shown are representative of at least three independent experiments. Integrated density values were normalized to untreated controls. Statistical significance is indicated (** *p* < 0.01, compared with antigen-treated cells).

Treatment with 5-*n*-nonadecylresorcinol, 5-*n*-heneicosylresorcinol, pinellic acid or tachioside (100–200 μM) inhibited antigen-induced degranulation and TNF-α expression in a dose-dependent manner (Figure 4b–e and Figure 5). 5-*n*-Nonadecylresorcinol and 5-*n*-heneicosylresorcinol were shown to have stronger inhibitory activity in antigen-induced degranulation than pinellic acid and tachioside. These results warrant further studies on the pharmacological activity and molecular mechanism of the compounds for the treatment and management of allergic diseases.

## 3. Discussion

Wheat bran (WB), which is a by-product in the process of milling, has been used as a food ingredient or a prebiotic. Although some side effects of WB-derived gluten, fructan and phytic acid to human health have been reported, WB is appreciated as a potential source for nutraceutical functions [10,11,12,13].

Dysregulated activation of mast cells and basophils is closely associated with the incidence and progression of allergic diseases. Allergen-specific IgE-FcεRΙ signaling activation in mast cells results in the release of various allergic and inflammatory mediators including histamine, TNF-α, and interleukins [5,6,18]. Therefore, the identification and regulation of key targets in IgE-FcεRΙ signaling pathways might be the effective approaches to the treatment of allergic diseases.

**Figure A1:**
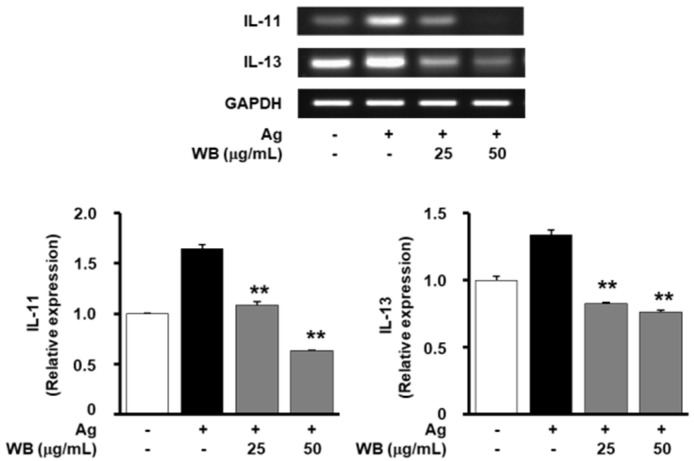
The levels of IL-11 and IL-13 in WB-treated cells. Cells were pretreated with WB (25, 50 μg/mL) for 20 min, followed by antigen stimulation for 20 min. The levels of IL-11 and IL-13 were determined by RT-PCR. Results shown are representative of at least three independent experiments. Integrated density values were normalized to untreated controls. Statistical significance is indicated (** *p* < 0.01, compared with antigen-treated cells).

In the present study, it is demonstrated that WB inhibits mast cell-mediated allergic responses in vitro and in vivo. The mechanism of these anti-allergic activities of WB involves inactivation of MAPK-dependent signaling pathways and down-regulation of allergic and pro-inflammatory mediators including TNF-α, COX-2 and iNOS (Figure 2). In addition, WB inhibited the expression of IL-11 and IL-13, which are associated with IgE-mediated allergic responses (Figure A1) [25,26]. Although the molecular mechanisms and targets of WB in the regulation of allergic responses remain to be further identified, this study demonstrate that the key signaling targets of WB are ERK and p38^MAPK^ (Figure 2). Furthermore, WB markedly inhibited IgE/antigen-induced local allergic reaction and compound 48/80-induced systemic anaphylaxis in vivo (Figure 3, Table 1), suggesting beneficial effects of WB as a potential coadjuvant in the treatment and management of allergic disorders. Finally, 5-*n*-nonadecylresorcinol, 5-*n*-heneicosylresorcinol, pinellic acid or tachioside isolated from the water extract of WB exerts inhibitory activities in antigen-induced degranulation and TNF-α expression (Figure 4 and Figure 5). Inhibitory activity of 5-*n*-heneicosylresorcinol in regulating degranulation and TNF-α expression was shown to be more potent than other compounds tested. In conclusion, these findings demonstrate the WB inhibits allergic responses in vitro and in vivo, and warrant further studies regarding the pharmacological activity and active ingredients in the design of specific inhibitors for the treatment and management of allergic diseases.

## 4. Materials and Methods

### 4.1. Reagents

2,4-Dinitrophenol (DNP)-specific IgE (D8406), DNP-conjugated bovine serum albumin (DNP-BSA, #A6661) and compound 48/80 (#C2313) were obtained from Sigma-Aldrich (St. Louis, MO, USA). Antibodies were purchased from commercial sources: anti-phospho-extracellular signal-regulated kinase (p-ERK, #4370) and anti-phospho-p38 mitogen-activated protein kinase (p-p38^MAPK^, #9211) (Cell signaling, Beverly, MA, USA); anti-actin (Sigma-Aldrich, #A2066); anti-cyclooxygenase-2 (COX-2, #sc-1747), anti-inducible nitric oxide synthase (iNOS, #sc-7271) and anti-IgG horseradish peroxidase conjugates (Santa Cruz Biotechnology Inc., Santa Cruz, CA, USA).

### 4.2. Preparation of the Water Extract of Wheat Bran

Wheat bran (WB) was obtained from a local milling plant (Milex Biotech Co., Gwangju, Korea). The bran was milled and passed through a 0.5 mm sieve. A voucher specimen (G36) was deposited in the Herbarium of the Bio-center, Gyeonggido Business & Science Accelerator (GBSA), Suwon, Korea. Wheat bran powder (0.5 kg) was extracted with 5 L of distilled water at a temperature from 80 to 100 °C in reflux for 3 h to give a water extract. After cooling to room temperature and then filtering (Advantec No. 2, Toyo Kaisha Ltd., Tokyo, Japan), the water extract was concentrated under vacuum below 40 °C and weighed to determine the yield (5 g). The extracts were completely dried in a freeze-drier and stored at −20 °C until further use.

### 4.3. Chromatographic Separation of the Water Extract of Wheat Bran

5-*n*-Nonadecylresorcinol, 5-*n*-heneicosylresorcinol, pinellic acid and tachioside from the water extract of WB were isolated and identified as described previously [21]. Conditions for High performance liquid chromatography (HPLC) were described as follows: Waters Alliance e2695 separating module (Waters Co., Milford, MA, USA) using photodiode array detector (Waters 2998) with autosampler and column oven was used for the analysis. Separation was achieved using a Kromasil 100-5-C18 column (5 µm, 250 × 4.6 mm i.d., AkzoNobel, Bohus, Sweden). The mobile phase consisted of water-trifluoroacetic acid (99.95:0.05; *v*/*v*) (solvent A) and acetonitrile (solvent B). The elution was performed using the following gradient: initial 95:5 (A:B *v*/*v*); 10 min 95:5 (A:B *v*/*v*); 30 min 50:50 (A:B *v*/*v*); 45 min 0:100 (A:B *v*/*v*); and 70 min 0:100 (A:B *v*/*v*). The mobile phase flow rate was 1.0 mL/min. The injection volume was 10 μL, and the column temperature was set at 30 °C. All operations, including data acquisition and analysis, were controlled by Empower™ 3 chromatography data software (Waters Co., Milford, MA, USA). The HPLC chromatogram of the water extract of WB was presented in Figure 4a.

### 4.4. Cell Culture Conditions

Rat basophilic leukemia (RBL)-2H3 cells were purchased from the American Type Culture Collection (Manassas, VA, USA). Cells were grown in 15% fetal bovine serum-Eagle’s minimum essential medium with 100 units/mL penicillin and 100 μg/mL streptomycin (Thermo Fisher Scientific, Waltham, MA, USA) at 37 °C in a humidified atmosphere containing 5% CO_2_.

### 4.5. Animals

Six-week-old male Balb/c mice and Sprague-Dawley rats were purchased from Orient, Inc. (Seoul, Republic of Korea). During the experiment, animals were housed in polycarbonate cages and acclimated to standard conditions of 12 h light/dark cycle, 20 ± 2 °C, and relative humidity of 45 ± 10% in a specific pathogen-free environment. The animal studies were performed in accordance with AAALAC-international guidelines and repeated at least three times using five rats (IgE-induced passive cutaneous anaphylaxis) or mice (compound 48/80-induced systemic anaphylaxis) per group in each experiment. The experimental protocol had received prior approval from the Institutional Animal Care and Ethical Use Committee of the Gyeonggido Business & Science Accelerator (Approval No. 2018-10-0003).

### 4.6. Cytotoxicity Assay

Cell viability was determined using 3-[4,5-dimethylthiazol-2-yl]-2,5-diphenyltetrazolium bromide (MTT) assay. RBL-2H3 cells in 96-well plates (1 × 10^4^ cells/well) were treated with different concentrations (25–100 μg/mL) of WB extract for 24 h. Following culture for 24 h, 0.1 mL of MTT solution (5 mg/mL) was added into each well and further incubated for 2 h. After the supernatant were removed, 0.1 mL of dimethyl sulfoxide (DMSO) was added to dissolve the purple formazan and the plate was rocked for 10 min. The absorbance was measured using a microplate reader (SpectraMax 340PC, Molecular Devices, San Jose, CA, USA) at 540 nm.

### 4.7. Stimulation and Measurement of Degranulation in RBL-2H3 Cells

RBL-2H3 cells were transferred to 24-well plates (5 × 10^5^ cells/well) and sensitized with DNP-specific IgE (50 ng/mL) for 4 h at 37 °C in a humidified atmosphere containing 5% CO_2_. The cells were washed and incubated with piperazine-*N*,*N*′-bis (2-ethanesulfonic acid) (PIPES) buffer (pH 7.2) containing 5.6 mM glucose, 1 mM calcium chloride and 0.1% BSA for an additional 10 min at 37 °C. Next, the cells were pretreated with or without WB extract for 20 min prior to the antigen DNP-BSA (25 ng/mL) stimulation for 20 min. Degranulation was determined by measuring the activity of β-hexosaminidase, a granule marker protein, as described previously [27]. The supernatant was transferred into a 96-well plate and incubated with substrate (1 mM *p*-nitrophenyl-*N*-acetyl-β-d-glucosaminide) for 1 h at 37 °C. The reaction was stopped by adding 0.1 M sodium carbonate/sodium hydrogen carbonate buffer. The levels of *p*-nitrophenol were measured using a microplate reader at 405 nm. The results were expressed as the percentage of maximal antigen-stimulated degranulation.

### 4.8. RNA Extraction and Reverse Transcriptase-Polymerase Chain Reaction (RT-PCR)

RBL-2H3 cells in 6-well plates (1 × 10^6^ cells/well) were incubated with DNP-specific IgE (50 ng/mL) for 4 h. After washing, cells were pretreated with WB extract or WB-derived compounds for 20 min and followed by DNP-BSA (25 ng/mL) stimulation for 20 min. Total RNA was extracted using Trizol^®^ Reagent, and was reverse-transcribed by the Superscript^®^ first-strand synthesis system (Thermo Fisher Scientific). Polymerase chain reaction was performed at 95 °C for 30 s, at 60 °C for 30 s, and at 72 °C for 90 s, for 30 cycles. The primer sequences for PCR were as follows: tumor necrosis factor-α (TNF-α), forward 5′-CACCACGCTCTTCTGTCTACTGAAC-3′ and reverse 5′-CCGGACTCCGTGATGTCT AAGTACT-3′; interleukin-4 (IL-4), forward 5′-ACCTTGCTTCACCCTGTTC-3′ and reverse 5′-TGAGCGTGGACTCATTC-3′; IL-11, forward 5′-TTGGTACTTGGAGCGG-3′ and reverse 5′-CACTGTGAATAGACTTCGT-3′; IL-13, forward 5′-ACAGCTCCCTGGTTCTCTCA-3′ and reverse 5′-CCCCCATTCACTACACATCA-3′; glyceraldehyde-3-phosphate dehydrogenase (GAPDH), forward 5′-GAGTCAACGGATTTGGTCGT-3′ and reverse 5′-GACAAGCTTCCCG TTCTCAG- 3′. Bands were imaged using a ChemiDoc™ XRS system (Bio-Rad Laboratories) and quantified using Quantity One software, version 4.6.3 (Bio-Rad Laboratories, Hercules, CA, USA).

### 4.9. Western Blot Analysis

RBL-2H3 cells in 6-well plates (1 × 10^6^ cells/well) were incubated with DNP-specific IgE (50 ng/mL) for 4 h. After washing, cells were pretreated with WB extract for 20 min and followed by DNP-BSA (25 ng/mL) stimulation for 20 min. Cells were rinsed twice with ice-cold phosphate-buffered saline (PBS, pH 7.4) and lysed for 30 min at 4 °C by incubation in lysis buffer (50 mM Tris-HCl, pH 7.5, 0.15 M NaCl, 1% NP-40, 0.1% SDS, 1 mM DTT and 1 mM PMSF) with protease inhibitor cocktail (Sigma-Aldrich). Cell lysates were centrifuged at 12,500× *g* for 20 min at 4 °C, and the supernatants were subjected to SDS-PAGE and western blot analyses as described previously [28]. All western blot analyses are representative of at least three independent experiments.

### 4.10. IgE-Induced Passive Cutaneous Anaphylaxis (PCA)

DNP-specific IgE (0.5 μg) was intradermally injected into rat dorsal skin. After 48 h, WB extract (50–200 mg/kg body weight) was orally administered 1 h prior to intravenous injection with 1 μg of the antigen DNP-BSA containing 4% Evans blue. Following the antigen stimulation for 30 min, the treated skin tissues were removed to measure the amount of dye extravasated by the antigen. The absorbance of the dye was measured at 620 nm, and the amount of dye was calculated by using a standard curve of Evans blue.

### 4.11. Compound 48/80 Induced Systemic Anaphylaxis

Anaphylactic shock in mice was induced by intraperitoneal injection of a mast cell degranulator compound 48/80 (8 mg/kg body weight). WB extract (50–200 mg/kg body weight) was orally administered 1 h prior to injection of compound 48/80. Mortality (%) within 1 h following the compound 48/80 injection was presented as the percentage of dead mice of total experimental mice.

### 4.12. Histamine Release Assay

Histamine levels in blood were determined using a fluorometric method. Reaction of histamine with *o*-phthalaldehyde at alkaline pH was terminated by acidification with hydrochloric acid. Fluorescence intensity was measured at 360/440 nm with a multi-label microplate reader (VICTOR3, PerkinElmer Life and Analytical Sciences, Shelton, CT, USA).

### 4.13. Statistical Analyses

Statistical analysis was performed using student’s t test. Results are presented as the mean ± standard error of the mean (SEM) from at least three independent experiments and considered to be statistically significant when *p* < 0.05.

## Figures and Tables

**Table 1:** Effect of WB on compound 48/80-induced mortality.

Treatment	Compound 48/80 (8 mg/kg)	Mortality (%)
Untreated	+	100
WB (50 mg/kg)	+	100
WB (100 mg/kg)	+	60
WB (200 mg/kg)	+	0
